# Sanitary Convention at Albion, Michigan

**Published:** 1888-09

**Authors:** 


### SANITARY CONVENTION AT ALBION, MICHIGAN.

WATER SUPPLY.

Professor VICTOR C. VAUGHAN, M. D., of Ann Arbor, Michigan, in an address, said: I suppose, if we were supplied with pure air, unadulterated food, and uncontaminated water, there would still be room for doctors and undertakers, but disease would be greatly decreased, suffering lessened, and life prolonged. It is a law applicable to all creatures, that when compelled to live upon their own excreta they sicken and die. This is true of the most minute organism. It is true of man.

The most prominent of English historians has said: "The only real knowledge is that which can be converted into practical power. It is the only knowledge that has life and growth in it. All else dries up like the rain drop off the stones, or hangs like dust about the brain." Let us make this discussion as practical as possible.

There are three different sources from which we can draw our drinking water—cistern water, surface water, and subterranean water. The purity of cistern water will depend on the atmosphere through which it falls, the roofs upon which it falls, and the cistern in which it is collected. It is more likely to be contaminated by the atmosphere in manufacturing districts than in Michigan. Great attention should be given to the roofs where leaves, branches of trees, and the excrement of birds are likely to accumulate. The only way to keep the cistern itself free from contamination is to build it so well that no water from the surrounding soil will be allowed to enter it. To do this the excavation should be one or two feet larger than the cistern. Several cases of typhoid fever have been known to come from the use of cistern water. In one case a cistern was used which was surrounded by numerous privy vaults. The cistern leaked, and if water can leak out it can leak in. What effect would it have to filter the cistern water? This depends upon circumstances. The ordinary filter is a delusion and a snare. Charcoal filters do not free organic matters. Organic matter collects in the filter and it becomes a hot-bed of disease.

The purity or impurity of surface water will depend on the atmosphere through which the water falls and the soil through which it flows. A few years ago I visited a city in this State to study the water supply. It was gathered from the hills, which are studded with stables, pig-pens, and all kinds of impurities. It was washed down into the ravine and then into a pond from which the supply was obtained. Typhoid fever prevailed in this locality to a great extent. Surface water may be very pure. This is true where it falls on a surface unpolluted, and where the volume of water is large. The great volume causes a dilution. Most large cities use surface water, and this has all degrees of purity and impurity. Detroit has a fairly pure supply from the lake. Some cities have suffered greatly. This is true of Philadelphia. During the Centennial Exposition the contents of the cesspools passed into the river, then down a short distance into the water supply, and there was a great prevalence of typhoid fever. Some suppose that flowing water will purify itself. There may be a grain of truth in this, but germs have been known to be carried a long distance and still continue poisonous. The citizens of Trenton and Wyandotte have been complaining of the contamination of their water supply by the city of Detroit. Garbage has been carried down the river, and Trenton and Wyandotte have suffered greatly from typhoid fever. It is probable that a large stream of sewage may be carried a long distance without being distributed, because of its different temperature and different specific gravity. This is illustrated by the Missouri and the Mississippi. The Missouri is muddy and still remains muddy after running into the Mississippi. This principle is also illustrated by the Gulf Stream, which retains its warm temperature and does not become admixed with the water of the ocean. So the probability is that if one city pours its sewage into the river, the city below will drink this sewage.

The purity of subterranean water depends on the purity of the soil through which it percolates. The popular idea is that percolating water purifies itself. This has been demonstrated to be a sad mistake. This is especially true of the sandy soil in this State. The distance organic matter will pass through a soil and still maintain its virulent properties is remarkable. (Dr. Vaughan here referred to experiments made in Richland, Kalamazoo County, showing that lithium sown over a proposed cemetery passed to a well thirty rods distant. He also stated how the germs of typhoid fever had been caried through a mile of porous soil in Lausanne, Switzerland.) Subterranean water will never be pure unless it passes through a pure soil. You can't have pure water as long as you have privy vaults and cesspools, and if there are any disease-germs in your soil they will find their way into your well.

It is known that cholera is spread by bad water. Where do we find the home of cholera? In India. Why do they have it in India, and why so constantly? Every few years the people of India hold festivals. They make pilgrimages a thousand miles on foot. The festivals occur in the hot months, just before the rains. They only eat meat that has been blessed, and after being blessed not a bit of it is thrown away. They are crowded in huts. They travel in small bands. They use the banks of rivers when obliged to obey the calls of nature. Disease and death make great havoc among them, and their corpses are buried in hills scooped in the sand. Cholera invariably breaks out, and the streams become contaminated with the cholera germs.

We shudder at the ravages of cholera, but let us come nearer home. Typhoid fever slays its hundreds where cholera slays its tens. Every death from typhoid fever is unnecessary. If the soil were not contaminated none would occur. Yet there are 1,000 deaths from this disease in Michigan each year; and for every one who dies ten persons are sick, and not a case is justifiable. Typhoid fever comes to those in the prime of life. Each patient is sick on the average about four weeks, and there are ten thousand sick during the year. This means not only a great loss of wages, but a nurse is required for each person sick, so that twenty thousand people give up their entire time to this disease four weeks out of each year. You may keep your premises clean, but only a little fence separates you from your neighbor, and he may be careless. If that neighbor should knock you on the head in a dark night he would be sent to prison, but he may contaminate your well, and rob you of your life and your family, and you do nothing. The time will come when it will be a criminal offense for any man, through carelessness, to give his neighbor a communicable disease.

During the past fall typhoid fever prevailed very extensively at Iron Mountain in this State. One of the physicians at Iron Mountain said that the people there were drinking the foulest water he ever knew people to drink. A sample of this water was sent me. After putting it in beef tea and keeping in an oven eight or ten days, the residue was injected in a cat's back, and the temperature rose in twelve hours from 99 to 104 degrees. This could not have been due to the cat's mind; it must have been due to the germ.

Some time ago a convention was held by the State Board in one of your neighboring cities, where the wells as a rule were only ten feet deep. The ground was honeycombed with cesspools. We earnestly pleaded with those in attendance at the convention that they would boil the water which they used. Yet in less than a month several who listened to the speeches were dead from typhoid fever. Do not regard what is said here as only talk. You are in danger of having typhoid fever so long as you have wells and privy vaults. Until a system of sewerage is introduced you should substitute for the privy vault the dry-earth closet. Next find a good water supply. I would advise you to employ some good civil engineer, pay him, and have him investigate the different sources of supply. Then decide where the water shall come from. Then get bids from different water companies. In Ann Arbor we paid a civil engineer two hundred and fifty dollars, and we now pay only forty-five dollars a hydrant.

### SCHOOL HYGIENE.

On this subject H. W. BRADLEY, of Albion, Michigan, said: The character of the citizens of the future will be determined, to a great extent, by the character and success of the schools of today. The success of the schools depends upon the physical as well as the mental and moral training of the pupils. The belief is quite general that the children of American families are physically declining, and "too much education" is frequently given as the principal reason for this degeneracy. This subject is one of vital importance, and should be discussed by school officers, that they may properly construct and arrange school buildings; by tax-payers, that they may realize full value for money expended for school purposes; by teachers, that they may care for the health of pupils; by physicians, that they may enlighten others less favored with opportunities for scientific research; by fathers, that they may make all necessary provisions; and by mothers, that they may perform their sacred duties acceptably. It is from a mother's standpoint that I look at this subject, and, though not expecting to instruct the members of this convention, I do hope to receive enlightenment from others.

*Ventilation.*—The selection of good sites for school buildings, and arrangements for proper ventilating, heating, and lighting them, are prominent features of this subject. The external beauty of the structures should be of small moment compared to these. One pleases the eye; the other affects the whole system. The subject of ventilation may receive all due attention in city schools, superintended, as they are, by educated men, and controlled by intelligent boards; but many school-houses in rural districts are very deficient in this particular. It is impossible for children to continue in perfect health any great length of time when they are compelled to endure several hours' confinement daily, in a room of unwholesome conditions. The air in our school-rooms (and homes also) should be able to stand the severest tests modern science can bring to bear upon them. The water should be above suspicion. The grounds and out-buildings ought to bear constant testimony to the doctrine that "Cleanliness is next to Godliness."

*High Buildings.*—The signs of the times seem to indicate a reform in the old custom of erecting school buildings many stories high, and then holding the more advanced classes in the higher rooms. The long train of ills following this practice is known only too well to the victims, mothers, and physicians. Welcome the reform, and may it reach every village in our State! The suggestion has been made by patrons that, in our smaller towns, ward school-houses be extended so that more grades may receive instruction there. This, of course, would exempt many of the younger children from the long, tedious walks necessitated by attending central schools.

*Recess or no Recess.*—I hesitate to criticise anything that receives the hearty support of some of the best teachers throughout our State, but the plea that comes from the mothers induces me to mention the subject. Mothers claim that individual recesses are extremely distasteful to timid children, and that they fail to avail themselves of the opportunities offered; that they often return from the school-room (after confinement during an entire session) with throbbing temples and feverish brow—nature's protest against the system. Some people think that we should go back to the old-fashioned plan of short, separate recesses. Others think that all should have a recess of at least ten minutes duration, where boys and girls and teachers may all go out of doors together and inhale the pure air of heaven, while windows and doors are thrown open in their absence. One writer says: "They would return from such a recess with blood made fresh and red, eyes and nerves rested, hunger appeased by a light lunch if desired, and nature's wants relieved; all would return to rooms cleansed of their foul exhalations by the flood of air pouring through them."

*Teachers.*—"The school teachers of today are the guardians of America's safety." And yet many of these teachers are young people, who have been rushed through a course of study and received the regulation diploma and certificate. They are often lacking in the maturity of mind and judgment necessary for the duties they assume—training children for future citizenship. Sometimes teachers are employed whose bodies are diseased and brains "muddled" by the use of narcotics. If it is our duty to examine the air in the school-rooms, it is a far *more imperative* duty that we understand the nature of the *moral atmosphere* of our teachers. Michigan now wisely decrees that every teacher shall know something about the science of health, and be prepared to teach the same. It is our privilege, as parents and patrons, to insist upon their making practical use of this knowledge. The better they understand sanitary science, and the more thoroughly they teach it, the better it is for the children. In order that we may *know* something about *how well* these matters are attended to in our schools, we would advise frequent visits to the school-rooms. The mind can not reach any great degree of successful development within a body composed of distorted bones, diseased nerves, compressed lungs, and bad blood.

*Food and Dress.*—Food and dress are important factors in the health of school children, and belong to the mother's kingdom. Teachers may suggest, physicians advise, fathers protest, but the mothers usually control. A wide field for reformation is here open for the labors of intelligent, Christian women. The requirements of health should never give place to the demands of fashion. The chest should have a good chance to expand, and the blood an opportunity to circulate as freely through the veins of our girls as of our boys. In winter we should procure for them mittens and overshoes, warm cloaks and hoods, thick shoes with low heels, and their forms should be enveloped with flannel. Rich and poor people alike should give their growing children substantial unadulterated food, with few, if any, dainties; supply them with milk, if possible, bread (graham is best), and ripe fruits in their season.

"Early to bed and early to rise" is as good a sentiment to teach the children of to-day as it was fifty years ago. We ought never to allow them to be long idle or unoccupied, and should expect them to perform tasks requiring manual labor. Let us teach the little ones the value of pure thoughts, and the danger of evil ones, which, fostered, lead to pernicious, ruinous habits. We can not hope that our children will not come in contact with evil, especially as in daily passing to and from school they meet with evil in one of its worst forms —intemperance—and alluring rooms are placed all along our public thoroughfares. That they may meet evil bravely and rise above it grandly is what we work and pray for.

After sowing all this good seed, and doing all of these very sensible things, may we not trust that their development will result in healthy, vigorous bodies, wherein the brains may live and grow. I know of nothing better to give our children than the glorious combination of industrious habits, healthy bodies, strong minds, and good morals.

*Discipline.*—Many of our schools are models of excellence in regard to discipline, and it would be idle to contend that discipline is unnecessary. The accumulation of useful, practical knowledge should be the aim of all school efforts, and this ultimate result ought never to be sacrificed for mere forms and ceremonies. Any discipline that might prove injurious to the health of pupils, is questionable. We would be sustained in censuring a teacher who would compel children to form out of doors, with wraps removed, for the purpose of entering the school-room in good order, unless the weather was perfectly warm and pleasant. Neither would it be unreasonable in cold weather to expect the teacher to superintend the matter of adjusting wraps for the very small children before they leave the school-house. You may say this is not the teacher's business, but it must be done if they endure the long walks, especially in the country, without suffering. If the teacher does not make it her business, who will?

*Overwork.*—The extensive course of study and consequent amount of mental work is often held responsible for all the ills that befall the children. This is as unjust as the cramming system is dangerous. If the brain, while in process of development, be overcrowded, there will be a corresponding weakening of the whole system. But our children can stand a goodly amount of hard study and deep thought if the laws of health are strictly observed in all details. Our country needs vigorous thinkers, but she will never find them among the children whose brains have been crowded with facts at the expense of the body. The gay-colored bird gives a lively effect to the jaunty hat on the head of a pretty girl, if we can banish the thought that for her adornment the little songster's heart was forever stilled, its musical voice forever hushed. Likewise a young Miss, bearing a diploma as the insignia of her successful pursuance of a course of study, is an interesting sight if her eye is bright, her step elastic, and complexion fresh; but if the honors have been secured at the sacrifice of health the spectacle is deplorable. Do not attribute failures in health to hard study alone. Remember the walks in the moonlight, the improper clothing and food, the hanging on gates, and lingering on piazzas, and then say whether the parents are not as responsible as the teachers. Many object to the competitive system as applied to our youths, and think it has produced a ruinous effect upon their mental as well as physical constitution. Allusion is seldom made to one great danger of this system. Scholars, in their desire to maintain class standing, are often compelled to work beyond their strength, and slight illness often results. Instead of removing the mental strain, and using a few hygienic precautions, physicians are sometimes summoned who prescribe stimulating medicines and quieting powders. You all know the result of the continuance of such a course—confirmed habits of daily doses of the dangerous drugs, until the body is wrecked and soul destroyed; and the victims are not the ones to be blamed. How carefully should the mother guard her children! How conscientiously should the physician who understands the danger, protect these young people, who do not. It would be better economy to pension the small army of physicians sent out from the medical colleges yearly than to support them by paying for professional services for our children, whose health may be *preserved* by giving attention to hygienic rules.

*Physiology and Hygiene.*—The legislature of our State has shown a deep interest in the citizens of the future, by making a law that "Instruction shall be given in physiology and hygiene, with special reference to the nature of alcohol and narcotics, and their effect upon the human system" in every department of every school, where public money is used in its support. "The district board shall require each teacher in the public schools, before placing the school register in the hands of the director, to certify therein whether or not instruction has been given in the school or grade presided over by such teacher." "And the director shall file with the township clerk a certified copy of such certificate." Any school board neglecting or refusing to comply with these provisions shall be subject to fine or forfeiture.

Any teacher of observation will sustain me in the assertion that boys who commence to use alcohol and tobacco will neglect their lessons and soon lose their ambition. Some men—habitual users of these poisons—have risen to eminent positions in life, but their success may not be a tithe of what it might have been had they been wise enough to have entirely relinquished the use of them.

It is a matter of surprise to teachers to find how readily pupils will learn and comprehend this study when it is properly explained. The wonderful network of telegraph lines called nerves which traverse the whole system, the miraculous organ which at every throb sends the life-giving blood into the most minute parts of the body, the great busy brain at work within the cranium, the intricate machinery of the digestive apparatus, the manner in which air penetrates the cells of the lungs, and then the deadly effects of alcohol and tobacco on all of these living tissues, are as well understood by children from five to ten years of age, as by men and women of middle life. A superintendent of schools in Massachusetts says: "This study ought to be taught. If the list of studies is already great, take something else out and make room for this, for it is entitled to the right of way."

Frances Willard says: "The life of a drinker of alcoholics has but two periods; in the first of which he could leave off if he would; in the last he would leave off if he could. How shall the young and thoughtless avoid this supreme peril of their youth, unless they know about it, and how shall they know unless they be taught, and how can instructors teach unless they have the proper authority?"

In the discussion of the subject, Professor V. C. VAUGHAN, M. D., said: The public schools are doing grand work and I do not believe that the course of study is too long. But I do believe that the hygienic conditions of the school-room are neglected; that children breathe bad air, and hence get headache. Brain-work in itself is not likely to hurt any one, provided the air is pure and fresh; but who can study when he is tired or diseased? The remedy is to make every teacher give some attention to sanitary science. The board of examiners should examine every teacher in hygiene, so that teachers would know about ventilation, etc. My observation has been that the school boards try to have everything right. Money is not spared to get the very best. Nearly every man has his own theory about ventilation, but the school board should employ some competent man who knows how to ventilate and heat. Children are often injured at home as well as at school. Children study at home, and by one defective lamp. It is said the schools ruin the scholars' eyes, but I believe that more damage is done by study at home. It is common to say that schools should not be built on hills, or more than two stories high—that climbing is bad. I hold that if it is properly done—if the scholar walks erect and bends only the knees in walking—it is good exercise.

### THE PREVENTION OF THE COMMUNICABLE DISEASES, FROM THE STANDPOINT OF THE STATE BOARD OF HEALTH.

HENRY B. BAKER, M. D., Secretary of the Michigan State Board of Health, in speaking of its work during the past fourteen years said: the board evidently believes that the "Prevention of the Communicable Diseases" is one of the most important measures which can engage its attention, and that work for this purpose is the most important that local boards of health can undertake. It is not difficult to imagine why this is true, because, by studying the vital statistics, it is found that the diseases which cause the greater proportion of the premature deaths in Michigan are the communicable diseases. Named in the order of their importance, the five diseases which cause most deaths in Michigan are: (1) consumption, (2) diphtheria, (3) pneumonia, (4) typhoid fever, and (5) scarlet fever. Excepting perhaps pneumonia, these are all communicable diseases; in common parlance, they are "catching"; not all of them are contagious, but all are in some manner spread from person to person. It thus appears not only that the communicable diseases have caused a large proportion of the premature deaths, but a large proportion of *all* the deaths, and that if we should prevent these communicable diseases we would change the character of our vital statistics—a much larger proportion of our people would die in ripe old age, a much larger proportion of the living inhabitants would be in the vigor of youth, and in the productive and enjoyable period of middle age; there would be prevented in each year in this State of Michigan, two or three thousand deaths of children from diphtheria and scarlet fever, and perhaps a thousand deaths of persons in the prime of life, from typhoid fever. Knowledge of these facts should prompt to greater efforts for the prevention of these diseases, and inasmuch as we now know how to prevent them, it seems strange that people do not uniformly act for their prevention. Probably the people would so act if they all knew that so large a proportion of the mortality is caused by these diseases, that they *are* preventable, and especially if all knew just *how* to prevent each one of these diseases.

These sanitary conventions do much to spread such information ; but they do not reach the people of the whole State fast enough, and the local boards of health, engaged as they are in the active work of restricting diseases which should have been prevented, and in other practical sanitary work, need the aid supplied by the different lines of work carried on by the State Board of Health, in order to spread that information which is essential to induce the people generally to sustain and co-operate with the local health authorities in restricting and preventing these diseases.

*Some of the Work of the State Board of Health.*—By means of reports from local health officers, physicians, and others, and in all practicable ways, the State Board collects information on these subjects; and in all practicable ways it seeks to spread this information where it will do the most good. It tries to have the information which it collects, and the best advice which it can give, placed before people at such times that it will be most likely to be accepted. Pamphlets, such as are distributed in this audience, containing concise statements of facts and directions how to restrict each one of these communicable diseases, are sent to localities where the diseases occur, to be distributed to the neighbors of the families in which the communicable disease occurs, because at such times of danger the neighbors will be most likely to read such pamphlets, and because after reading them they will then be most likely to co-operate with the local health officer for the restriction of the disease. Thousands of pamphlets on each of the most dangerous communicable diseases are distributed by the State Board. They are being distributed all the time. In order to distribute each kind of pamphlets in the localities where there are persons sick with the particular communicable disease of which that kind of pamphlet treats, it is necessary to know the localities in which each such disease occurs ; and this information to the office of the State Board is provided for by a law which requires every health officer in the State to keep the secretary of the State Board of Health constantly informed on this subject. There are in Michigan over 1,400 local boards of health—each one required by law to have a health officer. The law requires every householder and every physician who knows of a case of one of these dangerous diseases under his care to promptly notify the health officer or other officer of the local board of health. When these laws are faithfully obeyed, the local health officer can take prompt action for the restriction of the dangerous disease, the secretary of the State Board of Health can promptly supply pamphlets of instructions which the health officer can distribute to the neighbors, and frequently the central office can take other action which tends to aid the local officer in restricting the outbreak and preventing an epidemic ; and what is of even greater consequence, in so educating the people of that vicinity that thereafter they will be more ready to restrict that disease, and many of them be able to prevent outbreaks which otherwise would occur. Although this method of teaching the prevention of each of these diseases, while the disease is threatening, seems to be the best that is practicable for the State Board of Health, it seems too bad that it is not possible for the people of a locality to learn before a first outbreak occurs ; but something like experience or personal observation seems to be essential in order to awaken sufficient interest in the subject.

But while it is probably impossible to reach the adult population with facts and advice on these subjects faster than in some such way as it is being done by the methods just described, it certainly seems possible, so far as concerns the rising generation, to strike at the root of *this* ignorance which annually causes the death of thousands among us. This leads me to say that, in my opinion, *the Prevention of the Dangerous Communicable Diseases should be taught in the public schools.*

As that knowledge which tends *directly*

to the preservation of life is of more worth than that knowledge which tends indirectly thereto, the teaching of physiology and hygiene in the public schools as it is usually done may well be displaced by the teaching of the exact measures required for the prevention of those dangerous communicable diseases which cause the greater part of the deaths. Of course, if the teaching of physiology and hygiene as it is usually taught is of more consequence than some other studies, the displacement should be of such other studies; but the thought I wish to convey is that whatever is displaced it is of prime importance for the welfare of humanity that the exact principles of the prevention of those diseases which cause the most deaths should be taught in the public schools. It is as true now as when the Bible was written that "My people are destroyed for lack of knowledge."*

When the present methods of teaching "physiology and hygiene" were invented, *sanitary science* had not been evolved; but now we know approximately what diseases kill us, and we know how to prevent the most important of them. Is it not high time this knowledge were being utilized by the teachers throughout this country for the saving of the lives of those whom they are educating?

Here let me answer the question which some of you are wishing to ask—How do we know that we have that valuable knowledge directly useful for the saving of life? The answer is: Because whenever we act upon that knowledge the saving of life follows, and it follows so uniformly that we have come to believe that it is a result of such action. A few illustrations on a large scale are as follows: Computations, made and published a year or two since† show that since the State Board of Health was established, and the approved methods for the restriction and prevention of small-pox have been attempted. the deaths from small-pox in Michigan, returned to the office of the Secretary of State, have been much less than during the five years just before the board was established. Compared with that period, and making allowance for the increase of population, about 1,000 persons appear to have been saved from death from small-pox in Michigan during the eleven years, 1873–84. Similar computations indicate that about three thousand persons were saved from death from scarlet fever in Michigan during the same period of time.

Coming down to a more recent period of time, official reports from the health offices in Michigan, for the year 1886, have been compiled, under my direction, by Mr. George E. Willitts, whom many of you know as at one time a student at the college in this city. Among the pamphlets distributed in this audience you may see two diagrams which exhibit graphically some important facts worked out by Mr. Willitts in the office of the State Board of Health. From the diagram relating to scarlet fever, you may see that while in those outbreaks in which isolation or disinfection was neglected there was an average of nearly fourteen cases to one outbreak; in those outbreaks in which both these approved measures were enforced there was an average of less than three cases to an outbreak. Therefore, there were eleven cases to an outbreak saved by the more perfect methods, namely, by isolation and disinfection. That is, in the fifty-eight outbreaks in which the more perfect methods were enforced there was, compared with those of less perfect methods, a saving of 638 cases of sickness and forty-eight lives from this one disease in a single year. Probably the saving of life and health through efforts at the restriction and prevention of scarlet fever in Michigan in that year were many times what these figures show; but these figures supply a scientific basis for the comparison of two methods of action. The facts are obtained from the official reports of the health

*Hosea, Chap. III, 6.

† Proceedings Michigan State Board of Health, pp. 11--16; Annual Report Michigan State Board of Health, 1886, pp. 177--181.

officers in whose jurisdictions the outbreaks occurred, and they are reliable so far as relates to all outbreaks properly reported

The other diagram before you, relating to diphtheria in Michigan in 1886, shows even a greater saving of life and health through efforts for the restriction and prevention of that disease, there being an average of over sixteen cases to an outbreak in those outbreaks in which isolation or disinfection was neglected, while there were less than three cases to an outbreak in those outbreaks where both isolation and disinfection were enforced—a saving of over thirteen cases and over two deaths to each of the 116 outbreaks in which the more perfect methods were employed, compared with those outbreaks in which one important part of the work was omitted. This indicates that in the 116 outbreaks alone there were saved 298 lives and over 1,500 cases of sickness from diphtheria in Michigan in a single year by improved methods for its restriction and prevention.

These computations from official reports are submitted, not as indicating the total saving of life and health from diphtheria and scarlet fever in that year by such efforts as are recommended by the State Board of Health, but as supplying positive evidence that these diseases *can* be restricted and in great part prevented whenever the people and their officers coöperate promptly and fully in obeying the laws, and in carrying out the advice which is freely given by the State Board of Health.

If you ask what is that advice which the State Board of Health gives—what, in its opinion, are the best known measures for the restriction and prevention of each of the dangerous communicable diseases—I must say that the time at our disposal this evening will hardly permit of my stating the advice in full ; but it may be said that the methods are not the same for all diseases. Thus, the most important preventive of small-pox is vaccination; and small-pox, diphtheria, and scarlet fever are *contagious* diseases, and demand isolation of the sick and disinfection of the entire contents of the house, or at least of the room in which the disease has been ; while *typhoid fever* is probably *not contagious*, but demands the disinfection of all bowel discharges, and especial care for the protection of the purity of the drinking water. The spread of *consumption* may *probably* be lessened by a general attention to the disinfection of all the sputa of consumptives.

But for full, though concise statements, I must refer you to the several pamphlets which are published by the State Board of Health for gratuitous distribution.

